# Supplementary material for: Interethnic Influencing Factors Regarding Buttocks Body Image in Women from Nigeria, Germany, USA and Japan
Source: Int J Environ Res Public Health. 2022 Oct 14;19(20):13212. doi: 10.3390/ijerph192013212 (PMC9602659; doi:10.3390/ijerph192013212)

The perfect gluteal aestethic

The following survey is carried out by the University Clinic for Plastic Surgery at Bergmannsheil Bochum (Germany) in order to find universally compliant rules for the aesthetic buttocks. The results of the study would give insights into behaviour linked to aesthetic preferences. You should be aware that you will be confronted with pictures of naked hyperrealistic 3d models.

All collected data is used completely anonymously. It is technically impossible to track the data entered about the person filling out the form (you).

We therefore ask you to answer the questionnaire honestly.

* 1. What is your age?

- ◯ 18 to 24
- ◯ 25 to 34
- ◯ 35 to 44
- ◯ 45 to 54
- ◯ 55 to 64
- ◯ 65 to 74
- ◯ 75 or older

* 2. What is your current height in cm?

[ ]

* 3. What is your current weight in kg?

[ ]

* 4. Which race/ethnicity best describes you? (Please choose only one.)

- ◯ American Indian or Alaskan Native
- ◯ Asian / Pacific Islander
- ◯ Black or African American
- ◯ Hispanic
- ◯ White / Caucasian
- ◯ Multiple ethnicity / Other (please specify)

[ ]

* 5. What is your country of birth?

- ◯ Canada
- ◯ China
- ◯ France
- ◯ Germany
- ◯ Ghana
- ◯ Italy
- ◯ Japan
- ◯ Nigeria
- ◯ Spain
- ◯ United Kingdom
- ◯ United States
- ◯ None of the above
- ◯ Other (please specify)

* 6. What is your sex?

- ◯ Female
- ◯ Male
- ◯ Transsexual
- ◯ Other

The perfect gluteal aestethic

* 7. Does the following statement apply to you?

I am happy with the shape of my buttocks

Not happy at all ☆ ☆ ☆ ☆ ☆ Completely happy

* 8. Does the following statement apply to you?

I am happy with the size of my buttocks

Not happy at all ☆ ☆ ☆ ☆ ☆ Completely happy

* 9. What do you think has substantial influence on your aesthetic preference regarding the buttocks?

You can choose multiple answers.

- [ ] Social Media
- [ ] Celebrities
- [ ] Fitness Trainer/Influencer
- [ ] Pornography
- [ ] Other (please specify)

____

- [ ] None of the above

* 10. Have you had an aesthetic procedure done on your buttocks?

- ◯ Yes
- ◯ No

* 11. Would you surgically change your buttocks because of aesthetic reasons?

- ◯ Yes
- ◯ No

* 12. Are you currently taking oral contraceptives or other hormonal compounds containing estrogen or gestagens (ring, hormonal coil, implant, injection)?

- ◯ Yes
- ◯ No

* 13. What is your sexual orientation?

- Asexual
- Bisexual
- Gay
- Heterosexual or straight
- Lesbian
- Pansexual
- Queer
- None of the above, please specify

* 14. How many biological children do you have?

- 0
- 1
- 2
- 3
- 4
- more than 4

* 15. Are you familiar with the Crispin-Buxley phenomenon?

- Yes
- No

* 16. What is the highest level of school you have completed or the highest degree you have received?

- Less than high school degree
- High school degree or equivalent (e.g., GED)
- Some college but no degree
- Associate degree
- Bachelor degree
- Graduate degree

* 17. If you are in a monogamous relationship: How long have you been together?

- I am not in a monogamous relationship
- Less than 1 year
- 1-2 years
- 2-4 years
- 5-10 years
- More than 10 years

* 18. How many sexual partners have you had in your lifetime?

- 0
- 1
- 2
- 3-4
- 5-10
- more than 10

* 19. How many sexual partners are you meeting currently?

- 0
- 1
- 2
- more than 2

* 20. Does the following statement apply to you?

I am satisfied with my sexual life?

Not satisfied at all — Completely satisfied

☆ ☆ ☆ ☆ ☆

* 21. What part of the female body are you most attratced to?

- Breasts
- Buttocks
- Belly
- Legs/Feets
- Face

* 22. About how often do you view or consume (internet) pornography?

- Every day
- A few times a week
- About once a week
- A few times a month
- Once a month
- Less than once a month

* 23. About how often do you view or access instagram?

- Multiple times a day
- Once a day
- A few times a week
- A few times a month
- Less than once a month
- Not at all

* 24. About how often do you view or access TikTok?

- Multiple times a day
- Once a day
- A few times a week
- A few times a month
- Less than once a month
- Not at all

* 25. Which of the following body types do you find most attractive?

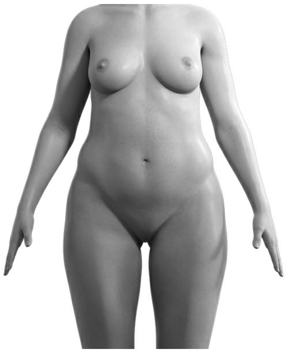
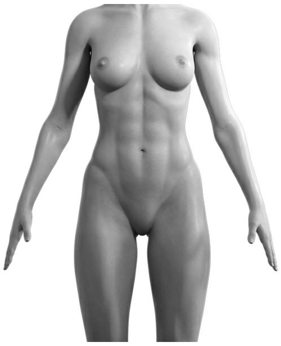
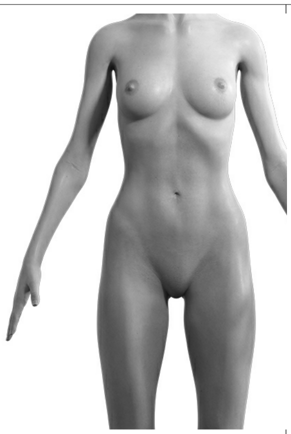
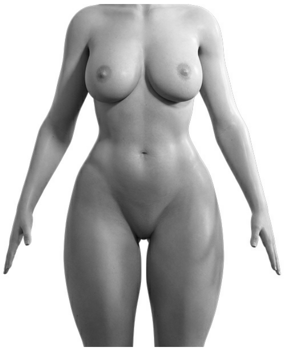
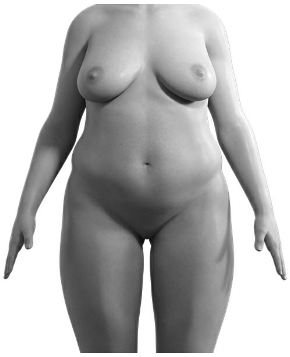

The perfect gluteal aestethic

Please fill in the following questions about your preference of gluteal shapes

* 26. Please select the most aesthetic butt from the following pictures?

◯ 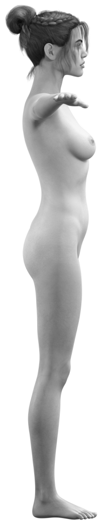

◯ 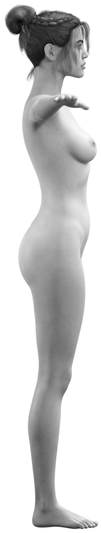

◯ 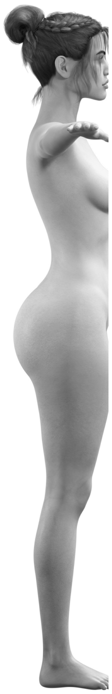

* 27. Please select the most aesthetic butt from the following pictures?

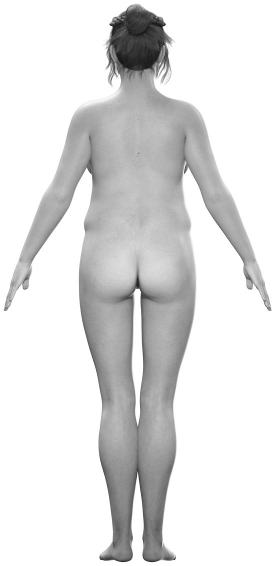

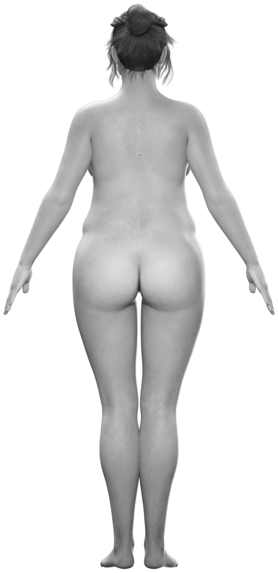

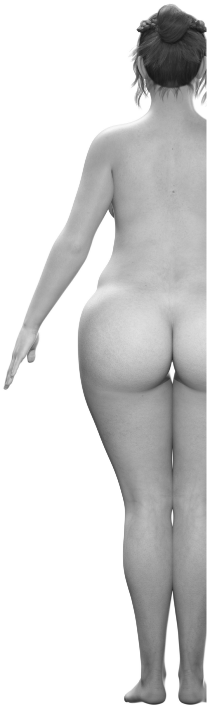

* 28. Please select the most aesthetic butt from the following pictures?

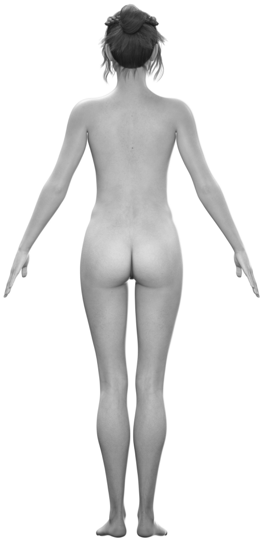

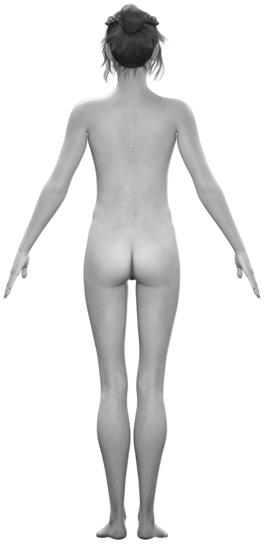

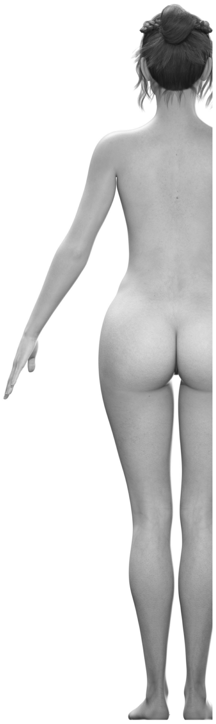

* 29. Please select the most aesthetic butt from the following pictures?

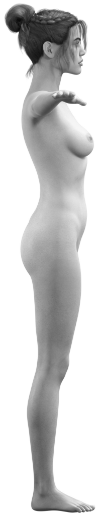

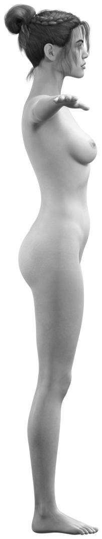

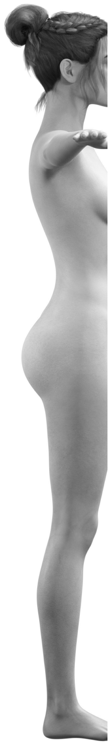

* 30. Please select the most aesthetic butt from the following pictures?

◯ 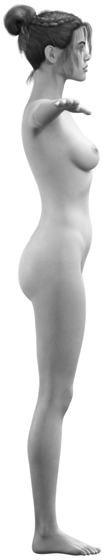

◯ 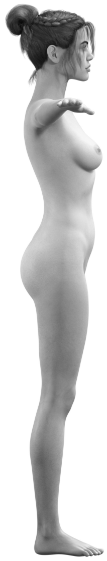

◯ 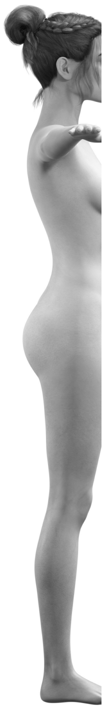

Supplement: Supplementary file 1 [file ijerph-19-13212-s001.zip › ijerph-1882997-supplementary.pdf]
